# The Role of Dentists and Primary Care Physicians in the Care of Patients with Sleep-Related Breathing Disorders

**DOI:** 10.3389/fpubh.2017.00137

**Published:** 2017-06-15

**Authors:** Harold A. Smith, Matthew Lee Smith

**Affiliations:** ^1^Dental Sleep Medicine of Indiana, Indianapolis, IN, United States; ^2^Department of Health Promotion and Behavior, College of Public Health, The University of Georgia, Athens, GA, United States; ^3^Department of Health Promotion and Community Health Sciences, School of Public Health, Texas A&M University, College Station, TX, United States

**Keywords:** sleep apnea, disordered breathing, dental, primary care, medical team, disparities

Obesity is a global epidemic that is well rooted in the United States and Western cultures (1–4). Recent attention of the magnitude of the epidemic warrants new approaches to health promotion, including expanded attention to obesity prevention by all health professions and disciplines. Driven by a combination of behavioral (e.g., unhealthy diets, physical inactivity) and environmental factors (e.g., food costs, community walkability, fast food establishment prevalence), obesity impacts individuals across the life-course and is responsible for morbidity, premature mortality, and escalating healthcare costs (5–8). The ramifications of obesity are pronounced among certain subpopulations, with disproportionate disparity and burden observed among racial/ethnic minorities, those of lower socioeconomic status, and those living in rural areas (9–13).

While the direct linkage of obesity to conditions such as diabetes, metabolic syndrome, and cardiovascular disease is well known (14, 15), these diseases may take years to manifest and be diagnosed. As such, there is growing recognition about more proximal risks associated with obesity, such as sleep-related breathing disorders (SRBD), including obstructive sleep apnea (16–19). When an individual struggles to breathe while sleeping, or actually stops breathing during sleep, additional stress is being placed on their heart, which can lead to health risks, including cardiovascular disease, pulmonary hypertension, enlarged heart, and stroke (20, 21).

Focusing on the signs and symptoms of obesity beyond body mass index (BMI) represents a health promotion approach with potential to prevent disease-related consequences by facilitating early screening and diagnosis. Such an approach may require new or slightly modified roles for existing healthcare professionals who are in regular contact with patients. While disease diagnoses are traditionally driven by physicians and nurse practitioners, opportunity exists for addressing the continuum of SRBD care within other disciplines during ongoing provider–patient interactions. Complementing specialized medicine efforts with additional professional expertise in SRBD can attenuate situations where patients go undiagnosed for long durations and experience exacerbated consequences once diagnosed because of advanced disease progression. Within this context, this article proposes a model that integrates dentists into multidisciplinary medical teams to collaboratively screen, refer, diagnose, and treat SRBD (see Figure 1).

**Figure 1:**
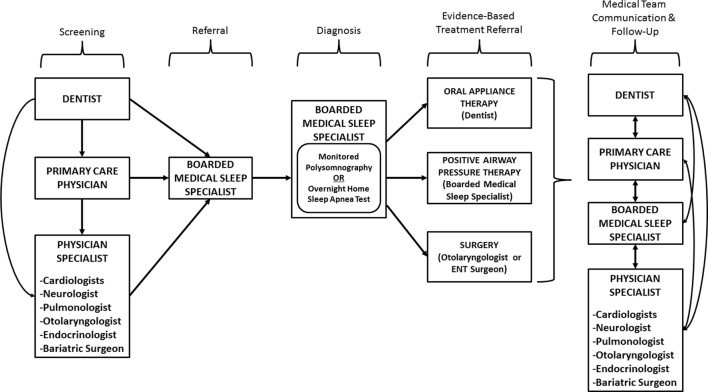
Involvement of a multidisciplinary medical team in the screening, referral, diagnosis, and treatment processes.

## Screening

Dentists and primary care physicians (PCP) are often the first line of detection and treatment for the public. Dentists potentially see their patients much more frequently than physicians, especially those who adhere to the recommended cleaning every 6 months. In addition, PCP potentially see their patients more frequently than do physician specialists (e.g., cardiologist, neurologist, pulmonologist, otolaryngologist), especially those who adhere to recommended annual checkups. Both dentists and PCP have the opportunity to discuss a variety of concerns that affect their patient’s quality of life, general well-being, and potential health risks. Since a great number of patients with undetected and untreated sleep disorders (such as obstructive sleep apnea) pass through the dental and PCP offices each day, it is necessary that these practitioners are educated about these conditions through trainings and continuing education courses so that they understand how best to embed practical assessments and referrals into routine care. Furthermore, their efforts can be twofold. The first is working with a referral system to link the patient to the proper specialist for diagnosis and to the proper professional for treatment. The second is to educate and consult the patient about healthy lifestyle choices that reduce risk factors for obesity and SRBD.

During a routine examination, the dentist can physically examine the patient’s teeth and perform an oral cancer screening that involves the tongue and the tissue in the back of the oral cavity. With minimum addition of time, these routine examinations can identify potential risk factors for SRBD such as sleep apnea. A dentist who has been educated about sleep apnea can also evaluate the patency of the airway, the size and position of the tongue, the size of the uvula, and the length of the soft palate tissue and tonsillar tissue. Following an examination of the oral tissues and noting the crowding of the oropharynx, dentists can weigh their patients and measure their height to determine their BMI, which can be used to facilitate discussions about obesity, its causes, and options for healthy eating and physical activity. Additionally, dentists could measure the neck circumference of their patients to assess SRBD risk (i.e., women with a 15-inch neck or greater and men with a 17-inch neck or greater are more likely to have sleep apnea) (22, 23). Further, dentists could take the patient’s blood pressure.

During routine office encounters, dentists and PCP can also collect subjective information from patients to assess their risk for sleep apnea. The most telling surrounds snoring. Loud snoring is caused by the tongue falling back into the airway and crowding with the uvula and soft palate and tonsils, if present. This crowding condition causes vibration of the tissue as patients breathe, and this vibration is heard as snoring. The crowding of the airway causing snoring can lead to a condition called upper airway resistance syndrome in which the struggling to breathe is more severe than snoring alone. Next on the continuum is mild sleep apnea, followed by moderate sleep apnea and then severe sleep apnea, which are all determined by the number of times per hour a patient completely or partially stops breathing. While everyone who snores does not necessarily have apnea, everyone with apnea snores. For this reason, snoring and apnea cannot be separated and both must be assessed/diagnosed. To assess snoring, patients should be asked: Do you snore? If yes, how would your ‘bed partner’ describe your snoring? Because apnea indicates that patients are not getting quality, restorative sleep during the night, follow-up questions include: When you wake up in the morning, do you feel refreshed? Are you tired or sleepy through the day? Do you have low energy throughout the day? Is it difficult to pay attention or remember things? Additional questions can be asked about the patient waking up through the night, going to the bathroom, having low libido, and experiencing diminished cognitive skills such as memory and concentration.

## Referral and Diagnosis

Routinely gathering physical and subjective screening information during office visits can arm dentists and PCP with information necessary to identify patients with increased SRBD risk and make referrals to other medical team members. Screening and referrals are among the most important roles dentists and PCP have because they routinely see a large number of patients (of all demographics) each year and can identify probable sleep apnea cases. Once identified, dentists and PCP can refer patients to boarded medical sleep specialists (i.e., board-certified by the American Academy of Sleep Medicine), who can then consult with the patient and order an overnight sleep test called a polysomnography (PSG) or an overnight home sleep apnea test (HSAT). The PSG is monitored by a sleep technologist at a sleep center, while the HSAT is self-conducted at the patient’s home and not directly monitored by a professional. Both methods are used by physicians to diagnose sleep apnea. These tests are used to objectively diagnose SRBD, including sleep apnea.

## Evidence-Based Treatment Referral

After sleep apnea is diagnosed by PSG or HSAT, the boarded medical sleep specialist can recommend multiple treatments. Possible treatments include weight loss, positional therapy (the best positions in which to sleep), continuous positive airway pressure (CPAP), surgery, and oral appliance therapy (OAT). Combination therapy is also common (e.g., CPAP/OAT, surgery/OAT, surgery/CPAP/OAT). The clinician or health professional responsible for overseeing each evidence-based treatment is different. Pending proper training and credentialing, dentists can effectively treat patients with sleep apnea using OAT (24–27). Regardless of the treatment or health professional overseeing that treatment option, patients should receive ongoing reinforcement about healthy behaviors during routine office visits.

## Communication and Follow-Up

Despite the treatment prescribed, ongoing communication among members of the multidisciplinary medical team is essential for success. A patient does not encounter just one healthcare professional; rather, they encounter a variety of professionals for different purposes at different times. As such, to establish an effective medical home for the patient, all healthcare professionals caring for the patient should be aware of (and involved with to some degree) the treatment decisions and related outcomes for that patient. By communicating treatment results with all medical team members, common and unified recommendations can be offered to the patient in terms of lifestyle modification, screening practices, and treatment adherence. Having multiple healthcare professionals repeatedly reinforce recommendations to the patient is preferable to less-effective unilateral messaging, which can be conflicting and cause confusion/misinterpretation.

Given the prevalence of obesity and associated disparities among subpopulations, additional efforts are needed to prevent its occurrence, delay the onset of associated conditions, and treat existing diseases caused by its presence. To complement the abundance of community-driven interventions to address obesity by modifying diet and physical activity (28–30), additional initiatives are needed in the clinical setting. While the costs to the healthcare system and society place emphasis on diseases such as diabetes and cardiovascular disease (31, 32), efforts to identify more proximal signs and symptoms of obesity-related conditions should not be overlooked. As such, dentists and PCP are uniquely positioned to screen patients for SRBD and sleep apnea because they are among the most frequently seen clinicians by the American population. In a ‘no wrong door’ to healthcare scenario, they can be the first encounter in a subsequent chain of healthcare interactions to prevent disease and improve health status. Dentists and PCP can be trained to perform physical and subjective screenings to identify sleep apnea risk and make referrals to boarded medical sleep specialists. Dentists can also guide and be included in sleep apnea treatments involving OAT. Regardless of the treatment received, dentists and PCP are valuable members of the medical team when caring for SRBD patients.

## Conclusion

The long-standing collaboration between dentists and physicians can help manage the obesity epidemic (and its related health conditions) by addressing SRBD through screening, diagnosis (by a physician), treatment, and referrals. Working together, dentists and physicians can fight the ramifications of sleep apnea and obesity to improve the health of individual patients and communities. This opinion indicates an ideal model of inter-professional care. However, challenges implementing such recommendations remain because of competing training demands, limited patient appointment times, and current reimbursement constraints. Thus, we call for additional efforts to increase awareness about this important public health and clinical issue. Studies are also warranted to examine the benefits and practicalities of implementing such a model in diverse settings. Ongoing efforts are needed to build upon the existing support given to the fields of medical and dental sleep medicine by organizations such as the American Medical Association, the American Dental Association, and insurance companies throughout the United States.

## Author Contributions

HS and MS conceptualized, drafted, and reviewed the manuscript.

## Conflict of Interest Statement

The authors declare that the research was conducted in the absence of any commercial or financial relationships that could be construed as a potential conflict of interest.
